# Synergic Effect between Adsorption and Photocatalysis of Metal-Free g-C_3_N_4_ Derived from Different Precursors

**DOI:** 10.1371/journal.pone.0142616

**Published:** 2015-11-13

**Authors:** Huan-Yan Xu, Li-Cheng Wu, Hang Zhao, Li-Guo Jin, Shu-Yan Qi

**Affiliations:** School of Materials Science and Engineering, Harbin University of Science and Technology, Harbin, P. R. China; SPECS Surface Nano Analysis GmbH, GERMANY

## Abstract

Graphitic carbon nitride (g-C_3_N_4_) used in this work was obtained by heating dicyandiamide and melamine, respectively, at different temperatures. The differences of g-C_3_N_4_ derived from different precursors in phase composition, functional group, surface morphology, microstructure, surface property, band gap and specific surface area were investigated by X-ray diffraction, Fourier transform infrared spectroscopy, scanning electron microscopy, transmission electron microscopy, X-ray photoelectron spectroscopy, UV-visible diffuse reflection spectroscopy and BET surface area analyzer, respectively. The photocatalytic discoloration of an active cationic dye, Methylene Blue (MB) under visible-light irradiation indicated that g-C_3_N_4_ derived from melamine at 500°C (CN-M500) had higher adsorption capacity and better photocatalytic activity than that from dicyandiamide at 500°C (CN-D500), which was attributed to the larger surface area of CN-M500. MB discoloration ratio over CN-M500 was affected by initial MB concentration and photocatalyst dosage. After 120 min reaction time, the blue color of MB solution disappeared completely. Subsequently, based on the measurement of the surface Zeta potentials of CN-M500 at different pHs, an active anionic dye, Methyl Orange (MO) was selected as the contrastive target pollutant with MB to reveal the synergic effect between adsorption and photocatalysis. Finally, the photocatalytic mechanism was discussed.

## Introduction

The global crises of environmental pollution and energy shortage have driven most scientists to research and develop novel techniques to both eliminate environmental pollution and utilize solar energy. In recent decades, photocatalysis has emerged from these techniques because of its utilization of solar energy for both organic contaminations degradation and hydrogen production [1]. Titanium dioxide (TiO_2_) has been regarded as one of the most prominent photocatalysts due to its non-toxicity, good stability and high activity [2]. However, as a semiconductor with wider band gap (3.0–3.2 eV), single phase TiO_2_ photocatalyst, such as anatase or rutile, can be excited only by the light in UV region [3], restricting the full use of solar resource. Although plentiful attempts of TiO_2_ modification, such as metal element doping, non-metal element doping, dye sensitization and heterojunction fabrication, have been made to extend its spectral response to visible light, the application aspects were still not satisfactory [4–6]. Thereby, several novel materials have been developed as the photocatalysts with visible light response, such as TaON [7], CaBi_6_O_10_ [8], AgBr [9], Ag_3_PO_4_ [10], BiOBr [11], BiOI [12], CdS [13], ZnO [14] and so on. These photocatalytic materials exhibit higher photocatalytic activity under visible light irradiation, but there still exist two main obstacles for their practical applications. On one hand, the presence of noble metal or transition metal in these photocatalysts makes the operational cost higher; on the other hand, some metal elements used are toxic and unsafe, which might be the latent hazards for the ecological environments.

In recent years, due to the unique physicochemical property and electronic band structure [15–19], graphitic carbon nitride (g-C_3_N_4_) has been developed as a metal-free and non-toxic photocatalyst with visible light response [20, 21]. g-C_3_N_4_ and g-C_3_N_4_-based materials have been widely investigated for photocatalytic hydrogen evolution, CO_2_ reduction, pollutants removal, organic syntheses and disinfection [22]. Furthermore, functionalized g-C_3_N_4_ has been applied in energy conversion and storage, such as fuel cells, electrocatalytic water splitting, supercapacitors and lithium ion battery [23]. g-C_3_N_4_ is considered to be the most stable allotrope among various carbon nitrides under ambient conditions [24]. The layered structure of g-C_3_N_4_ is like graphite, composed by two basic structure units: s-triazine and tri-s-triazine [25–27]. The π-conjugated graphitic planes in its structure are constructed by carbon and nitrogen atoms linked via sp^2^ hybridization [28]. Unlike the metal-containing photocatalysts that need expensive metal salts for preparation, g-C_3_N_4_ photocatalyst can be facilely prepared by thermal polycondensation of cheap precursors [29]. These precursors generally used are the carbon- and nitrogen-rich organic compounds, such as dicyandiamide [30], melamine [16, 31], urea [32], thiourea [33], cyanuric chloride [34], ethylenediamine with carbon tetrachloride [35], ammonium thiocyanate [36] and cyanamide [37]. For example, Dong et al employed a facile and eco-friendly approach to successfully fabricate nitrogen-rich g-C_3_N_4_ layered nanostructure by directly heating thiourea in air at 550°C and found that the sulfur species in precursor could chemically control the formation of g-C_3_N_4_ networks and accelerate the polymerization process of g-C_3_N_4_ [33]. Zhao et al developed a high-energy ball milling method to prepare g-C_3_N_4_ sample using cyanuric chloride and lithium nitride as precursors. The obtained product with atomic ratio of N to C was 1.23–1.30, similar to the theoretical stoichiometry of g-C_3_N_4_ [38].

As a metal-free polymeric photocatalyst with a narrower band gap of about 2.7 eV, g-C_3_N_4_ can directly realize the visible light response without any decoration and exhibit excellent photocatalytic activity for the degradation of organic compounds in water [34]. Compared with inorganic metal catalysts, this metal-free material has high chemical stability and can be tailored as desired, due to its polymeric properties, making it a promising photocatalyst in aqueous solution [39]. Yan et al reported that, when 1.0 g/L g-C_3_N_4_ synthesized from dicyandiamide was used as the photocatalyst, the degradation efficiency of Rhodamine B (RhB, 2×10^−5^ M) could reach about 100% after 100 min irradiation under a 300 W Xe lamp [16]. Cui et al employed a mesoporous g-C_3_N_4_ obtained from ammonium thiocyanate as the photocatalyst to degrade 4-chlorophenol (4-CP) in water. The experimental results indicated that the degradation efficiency of 1.2×10^−4^ M 4-CP could approach nearly 100% after 90min irradiation by visible light [36]. Ji et al reported that, after 3h irradiation by a 300 W Xe lamp, 2,4,6-trichlorophenol (2,4,6-TCP) with the concentration of 5×10^−5^ M could be completely degraded when 1g/L g-C_3_N_4_ synthesized from dicyandiamide was used as the photocatalyst [40]. However, to our best knowledge, the contrastive investigations for the photocatalytic degradation of different organic dyes by g-C_3_N_4_ derived from different precursors have not been systematically executed to reveal the synergic effect between adsorption and photocatalysis.

Hence, in this work, pyrolytic synthesis of g-C_3_N_4_ was conducted by heating dicyandiamide and melamine, respectively. The phase composition, chemical functional group, surface morphology, microstructure, surface property, band gap, specific surface area and surface Zeta potentials of the as-obtained samples at different conditions were studied by X-ray diffraction (XRD), Fourier transform infrared spectroscopy (FT-IR), scanning electron microscopy (SEM), transmission electron microscopy (TEM), X-ray photoelectron spectroscopy (XPS), ultraviolet-visible diffuse reflection spectroscopy (DRS), BET surface area analyzer and Surface Zeta potentiometer, respectively. An active cationic dye, Methylene Blue (MB), was used as the target pollutant to evaluate the photocatalytic activity of g-C_3_N_4_ samples obtained from different precursors. The affecting factors of the photocatalytic activity, including initial concentration of MB and photocatalyst dosage, were also analyzed in detail. Otherwise, an active anionic dye, Methyl Orange (MO), was employed as the contrast to investigate the photocatalytic behavior of g-C_3_N_4_ in the degradation of different dyes. Finally, based on the measurement of the surface Zeta potential of g-C_3_N_4_, the synergic effect between adsorption and photocatalysis was discussed.

## Materials and Methods

### 2.1. Synthesis method

g-C_3_N_4_ sample used in this work was synthesized by directly heating dicyandiamide and melamine at different temperatures, respectively. In brief, 6g of precursor powder were put into a crucible and heated to 460°C, 500°C, 540°C and 580°C with a heating rate of 20°C/min, respectively. Subsequently, the sample was kept at final temperature for 2h. After cooled, g-C_3_N_4_ sample was obtained and ground in a mortar for use. The g-C_3_N_4_ samples derived from dicyandiamide at 460°C, 500°C, 540°C and 580°C were labeled as CN-D460, CN-D500, CN-D540, and CN-D580, respectively. And, the samples obtained using melamine as the precursor at 460°C, 500°C, 540°C and 580°C were denoted as CN-M460, CN-M500, CN-M540, and CN-M580, respectively.

### 2.2. Characterization techniques

The phase composition of g-C_3_N_4_ was identified by XRD, recorded on a Rigaku D/max-3B X-ray diffractometer (Cu-K_α_ radiation, λ = 0.15418 nm) over the 2θ range of 10°-60° at 40 kV and 30mA. The chemical groups in g-C_3_N_4_ structure were confirmed by FT-IR, operated on a Nicolet Nexus infrared spectrometer after mixture of g-C_3_N_4_ sample with spectroscopic grade KBr (300 mg). The crystal morphology and microstructure of g-C_3_N_4_ were observed by SEM and TEM, realized on FEISirion200 scanning electron microscope and JEOL JEM-2010 transmission electron microscope, respectively. X-ray photoelectron spectroscopy (XPS) with Al Kα X-rays radiation (AXIS ULTRADLD, Kratos) was used to investigate the element compositions and surface properties of the samples. The binding energy was corrected using C1s (284.6 eV) as the internal standard. DRS spectra, recorded in the range of 400–800 nm, were implemented on an USB4000 UV-vis spectrometer (Ocean Optics) equipped with an integral sphere, using a standard template provided by South Africa Optics as the reference. The BET specific surface area of g-C_3_N_4_ was measured on a Sibata SA-1100 surface area analyzer, according to the nitrogen adsorption-desorption data at liquid nitrogen temperature. Surface Zeta potentials of g-C_3_N_4_ were examined by Zeta potential analyzer (Nano-ZS90) at different pH values.

### 2.3. Photocatalytic activity evaluation

Some grams of g-C_3_N_4_ powder were added to 100 mL organic dye solution with the concentration of 5 mg/L. Before irradiation by Xe lamp (simulated sunlight launcher with a power of 100 mW/cm^3^, wavelength range: 200–1000 nm), the suspension solution was stirred in dark for 45 min to reach the adsorption equilibrium. After that, Xe lamp was turned on and photocatalytic process was conducted in the next 75 min under the condition of continuous stirring. 5 mL of the suspension solution were taken out at each 30 min interval and centrifuged for 10 min to obtain the supernatant. The residual concentration of organic dye in the supernatant was measured by a 722-tpye UV-vis spectrophotometer at the maximum absorption wavelength of different dyes (MB: 665 nm; MO: 460 nm). The discoloration ratio of organic dye was calculated as follows: *D*(%) = (*C*_0_-*C*_t_)/*C*_0_×100%, where *C*_0_ is the initial concentration of organic dye and *C*_t_ the residual concentration of organic dye at reaction time *t*. Otherwise, some random tests were implemented at different conditions in order to check the repeatability of the experimental results.

## Results and Discussion

### 3.1. Characterization of g-C_3_N_4_ derived from different precursors

**Fig 1:**
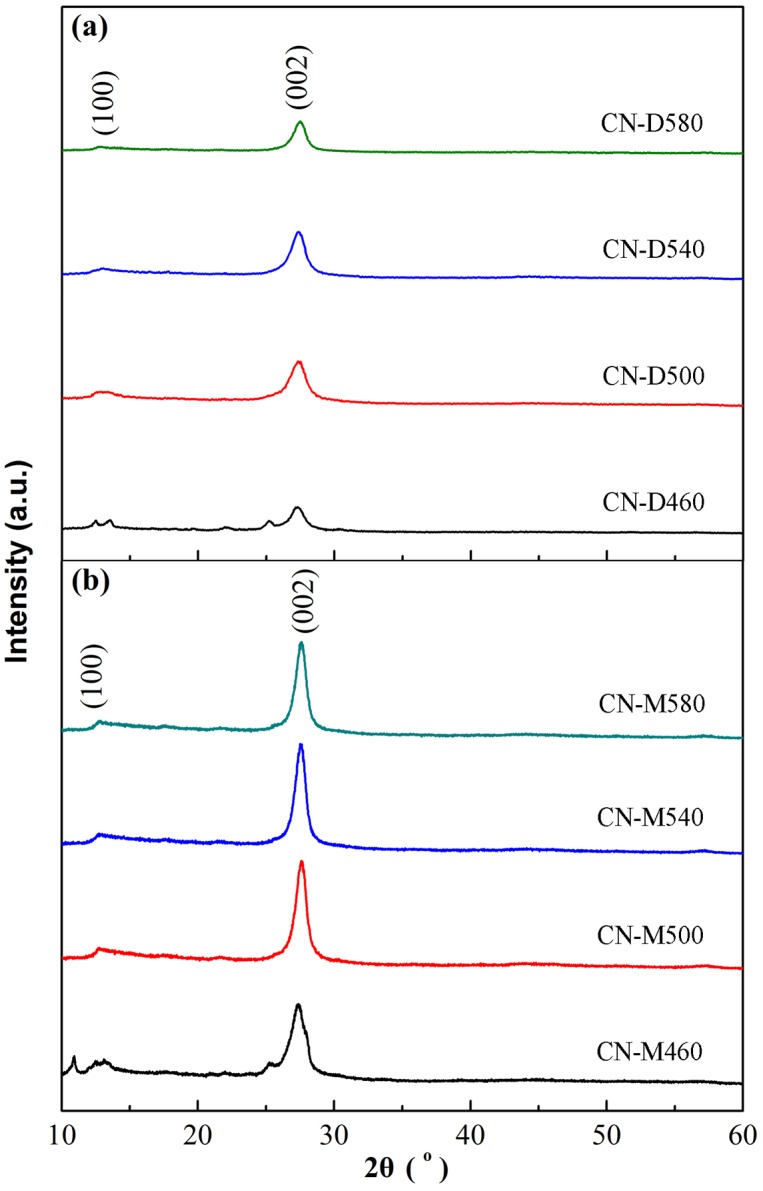
XRD patterns of g-C_3_N_4_ samples derived from different precursors at different temperatures. (a) Dicyandiamide. (b) Melamine.

**Fig 2:**
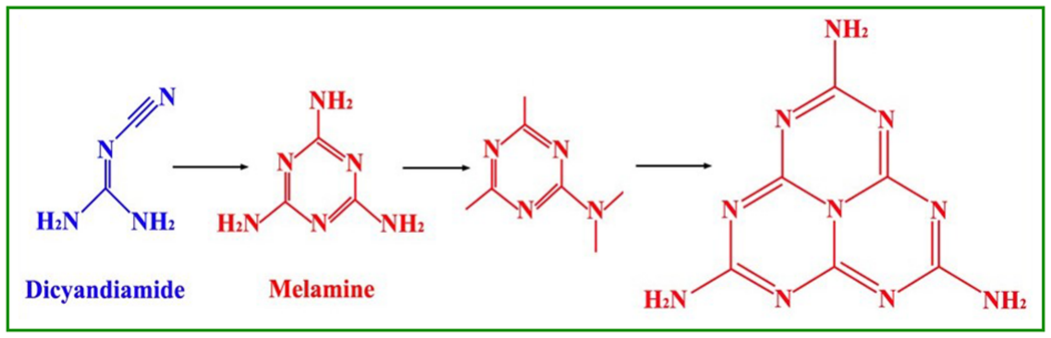
Polycondensation route for the formation of g-C_3_N_4_ from dicyandiamide and melamine.

XRD patterns of g-C_3_N_4_ samples derived from different precursors at different temperatures are presented in Fig 1(a) and 1(b), respectively, where it can be seen that there exist two distinct diffraction peaks for all the obtained samples. For g-C_3_N_4_ samples derived from dicyandiamide, these two diffraction peaks occur at 13.16° and 27.28°, respectively (Fig 1(a)). And, for the samples derived from melamine, the both peaks center at 13.05° and 27.59°, respectively (Fig 1(b)). Therefore, there is no difference in the position of XRD peaks for g-C_3_N_4_ samples prepared from different precursors, suggesting that all the samples possess the same crystal structure of g-C_3_N_4_. For the both peaks, the stronger one is generated by the stacking of the conjugated aromatic ring, indexed as the (002) crystal plane for graphite-like materials [41]; while, the weaker one is attributed to the in-plane ordering of tri-s-triazine units, assigned as the (100) crystal plane [42]. It also can be seen from the XRD patterns that, at the pyrolytic temperature of 460°C, two unknown impurity peaks occur near 11° and 25° for g-C_3_N_4_ samples derived from both dicyandiamide and melamine, which might be attributed to the incomplete polycondensation of precursors. Moreover, it should be noted that, at the same condition of temperature, g-C_3_N_4_ sample derived from melamine exhibits sharper XRD peaks than that from dicyandiamide, implying that the former has better crystallinity with less defects and disturbances in the graphitic structure. According to XRD results, the crystal size of g-C_3_N_4_ was computed by Scherrer formula *D* = *Kλ*/*β*cos*θ*, where *D* is the crystallite size (nm), *K* the Scherrer constant (about 0.9), *λ* the wavelength of Cu-K_α_ radiation (0.15418nm), and *β* the full width of (002) diffraction peak at half maximum [43]. The calculated crystallite sizes of all g-C_3_N_4_ samples are listed in Table 1. On the whole, at the same pyrolysis temperature, the crystallite size of g-C_3_N_4_ sample obtained from dicyandiamide is smaller than that from melamine. The possible explanation for this tendency might be that the polycondensation route for dicyandiamide to form g-C_3_N_4_ is different to that for melamine. According to previous reports [27, 44], these two routes are illustrated in Fig 2. The precursor melamine can be directly pyrolyzed and polymerized to form g-C_3_N_4_, nevertheless, dicyandiamide must be firstly condensed into melamine and then pyrolyzed and polymerized to form g-C_3_N_4_. The redundant step for dicyandiamide to form g-C_3_N_4_ encumbers the nucleation and growth of g-C_3_N_4_, thus it is not hard to understand that the crystallite size of g-C_3_N_4_ derived from dicyandiamide is smaller within the same pyrolysis time. Furthermore, for the same precursor, the crystallite size increases with the pyrolysis temperature increasing, suggesting that higher temperature favors the nucleation and growth of g-C_3_N_4_.

**Table 1:** Crystallite size, surface area and band gap of g-C_3_N_4_ derived from different precursors at different pyrolysis temperatures.

Sample	Crystallite Size (nm)	Surface Area (m^2^/g)	Band Gap (eV)
CN-D460	5.05	4.95	2.76
CN-D500	6.36	10.59	2.71
CN-D540	6.39	10.73	2.57
CN-D580	7.04	23.19	2.56
CN-M460	5.32	7.26	2.75
CN-M500	8.01	23.17	2.71
CN-M540	8.16	23.50	2.62
CN-M580	8.17	26.02	2.60

**Fig 3:**
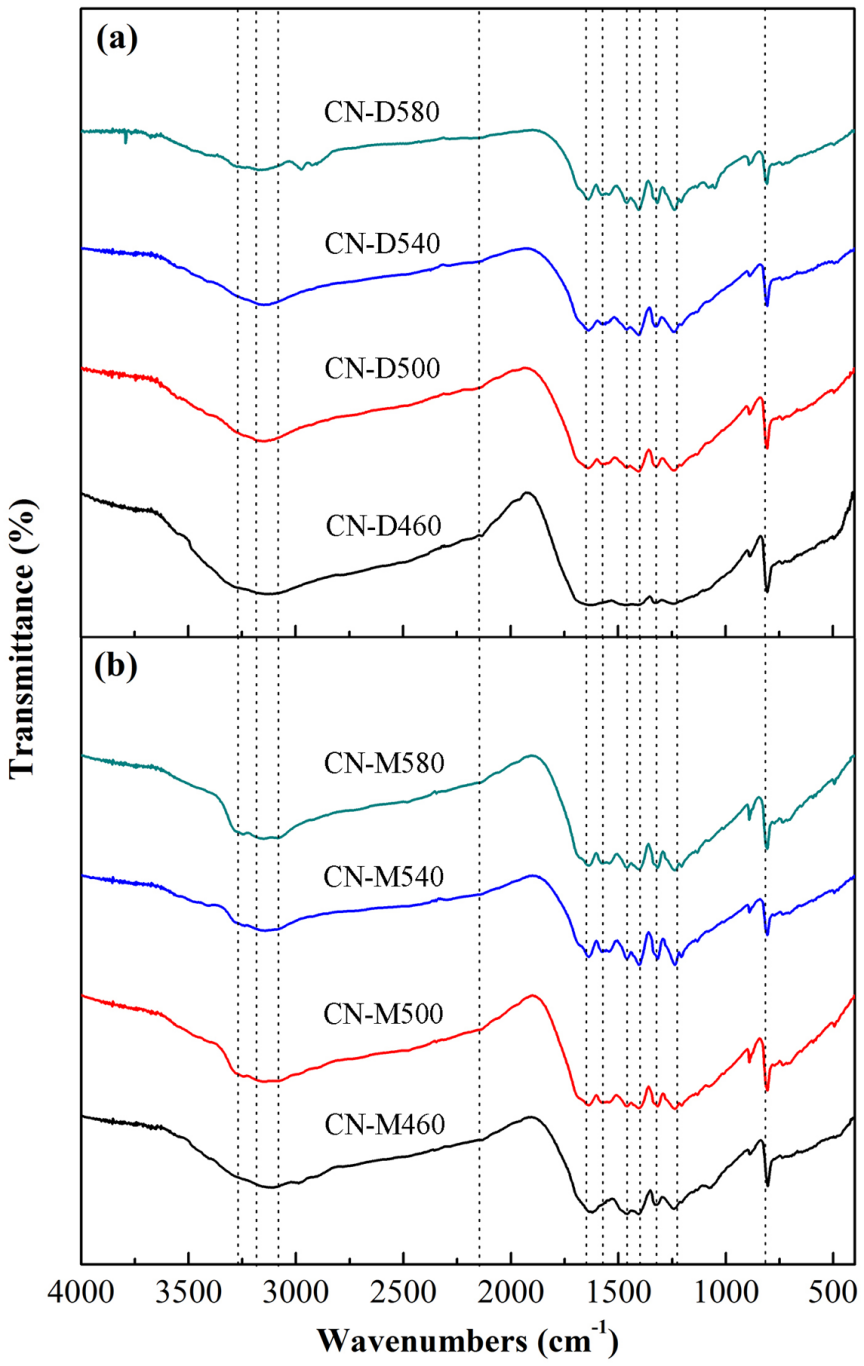
FT-IR spectra of g-C_3_N_4_ samples pyrolyzed from different precursors at different temperatures. (a) Dicyandiamide. (b) Melamine.

The FT-IR spectra of g-C_3_N_4_ samples pyrolyzed from different precursors at different heating temperatures are shown in Fig 3(a) and 3(b), respectively. All absorption bands in FT-IR spectra almost occur at the same position for g-C_3_N_4_ samples obtained under different conditions. The characteristic absorption bands at 1648.9 cm^-1^, 1571.8 cm^-1^, 1458.0 cm^-1^ and 1398.2 cm^-1^ are assigned to the stretching vibration modes of the carbon nitride heterocycle [45]. The sharp band observed at 808.1 cm^-1^ is corresponded to the bending vibration mode of triazine units [46]. The characteristic absorption bands at 1311.4 cm^-1^ and 1238.1 cm^-1^ are attributed to the stretching vibration modes of C-N(-C)-C or C-NH-C unit [47]. Meanwhile, the broad band near 3151.3cm^-1^ is possibly induced by the residual uncondensed amino component or O-H stretching vibration of absorbed H_2_O molecules [48]. The residual amino component in the as-obtained g-C_3_N_4_ sample mainly stemmed from the incomplete condensation of organic precursor during the pyrolytic process. Furthermore, this broad absorption band might also be associated with C-NH_2_ and 2C-NH bonds, generated by the linkage between residual hydrogen atoms and graphene-like C-N sheet edges [49]. And, there exists a very weak band at 2146.5cm^-1^ assigned to C-N, which implies that trace amounts of the precursor molecules were not cracked completely during the pyrolytic process [50].

**Fig 4:**
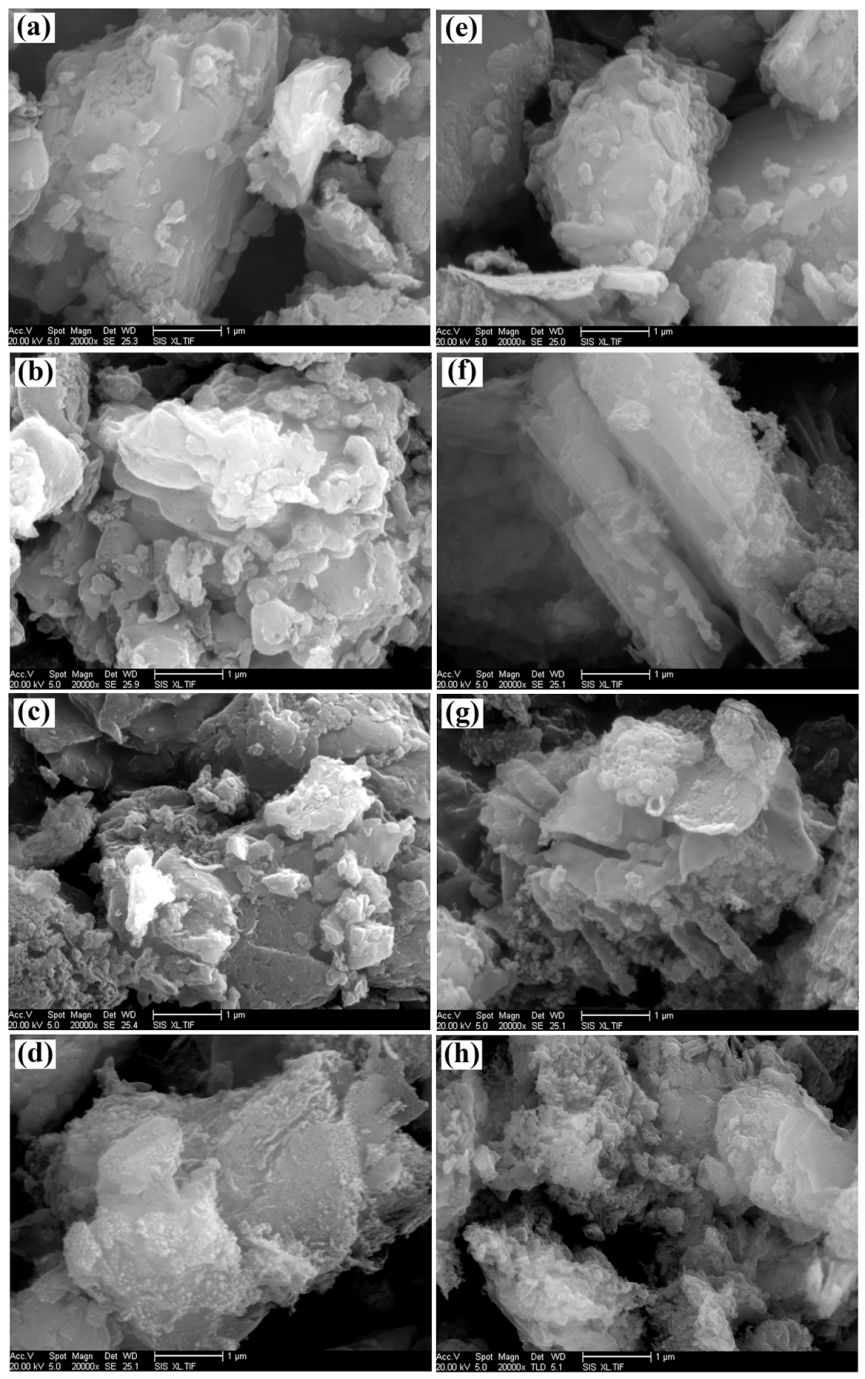
SEM images of g-C_3_N_4_ samples. (a) CN-D460. (b) CN-D500. (c) CN-D540. (d) CN-D580. (e) CN-M460. (f) CN-M500. (g) CN-M540. (h) CN-M580.

**Fig 5:**
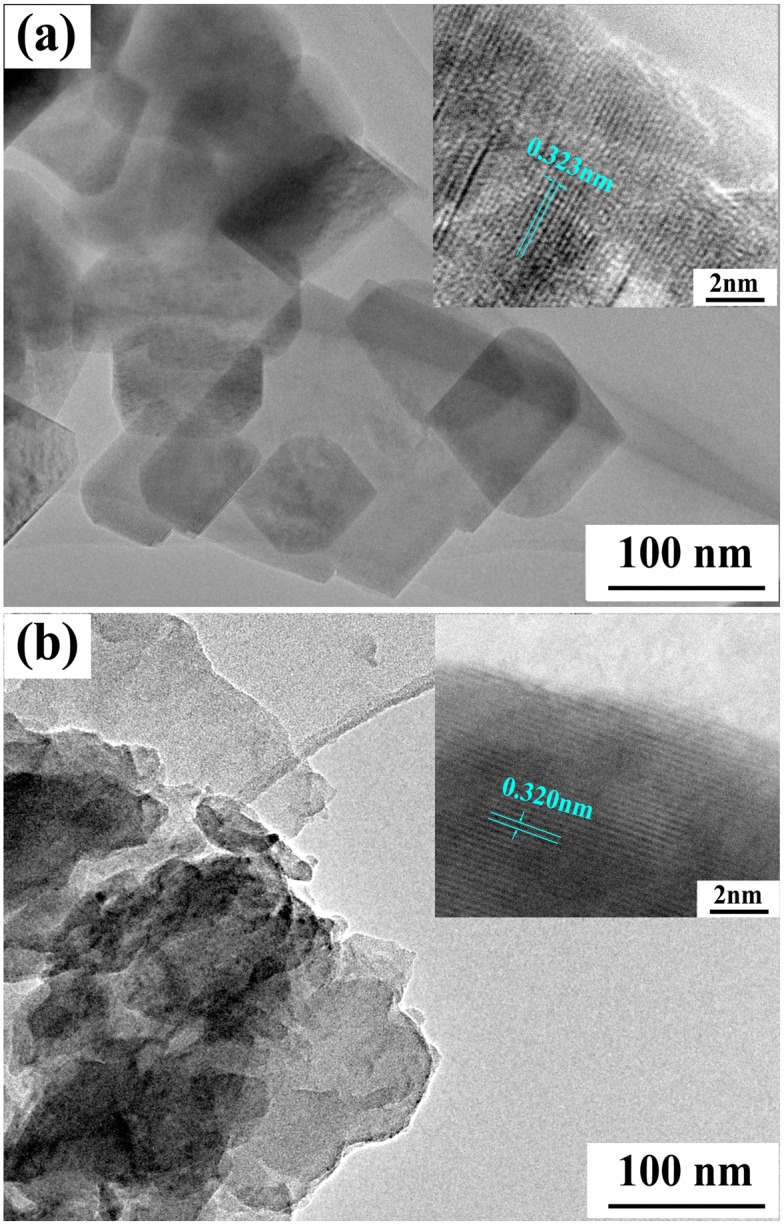
TEM and HRTEM (inset) images of g-C_3_N_4_ samples. (a) CN-D500. (b) CN-M500.

The SEM images of g-C_3_N_4_ samples derived from different precursors at different pyrolysis temperatures are shown in Fig 4, where it can be seen that all the g-C_3_N_4_ particles are in irregular shape and desultorily assemble together with the glassy morphology of typical g-C_3_N_4_, similar to the observations in other reports [38, 51]. There is no difference in the morphology for all these samples. The observed dimension of aggregated particles ranges from 1 μm to 5 μm, much larger than that calculated from XRD results. The possible reason for this situation might be that the calculated one is the size of a single crystallite; whereas, the observed one in the SEM micrograph is the size of the agglomerate composed by many g-C_3_N_4_ crystallites. Furthermore, TEM images of g-C_3_N_4_ samples obtained by directly heating different precursors at 500°C are shown in Fig 5(a) and 5(b), where the flat layered structure can be distinctly seen, indicating that g-C_3_N_4_ samples obtained from different precursors exhibit the same ordered structure with inter-layer stack. The lattice fringes observed in HRTEM images (see insets in Fig 5(a) and 5(b)) are assigned to (002) crystal plane. The crystal plane distance was determined as 0.323nm and 0.320nm for g-C_3_N_4_ sample derived from dicyandiamide and melamine, respectively, consistent with previous report [27].

**Fig 6:**
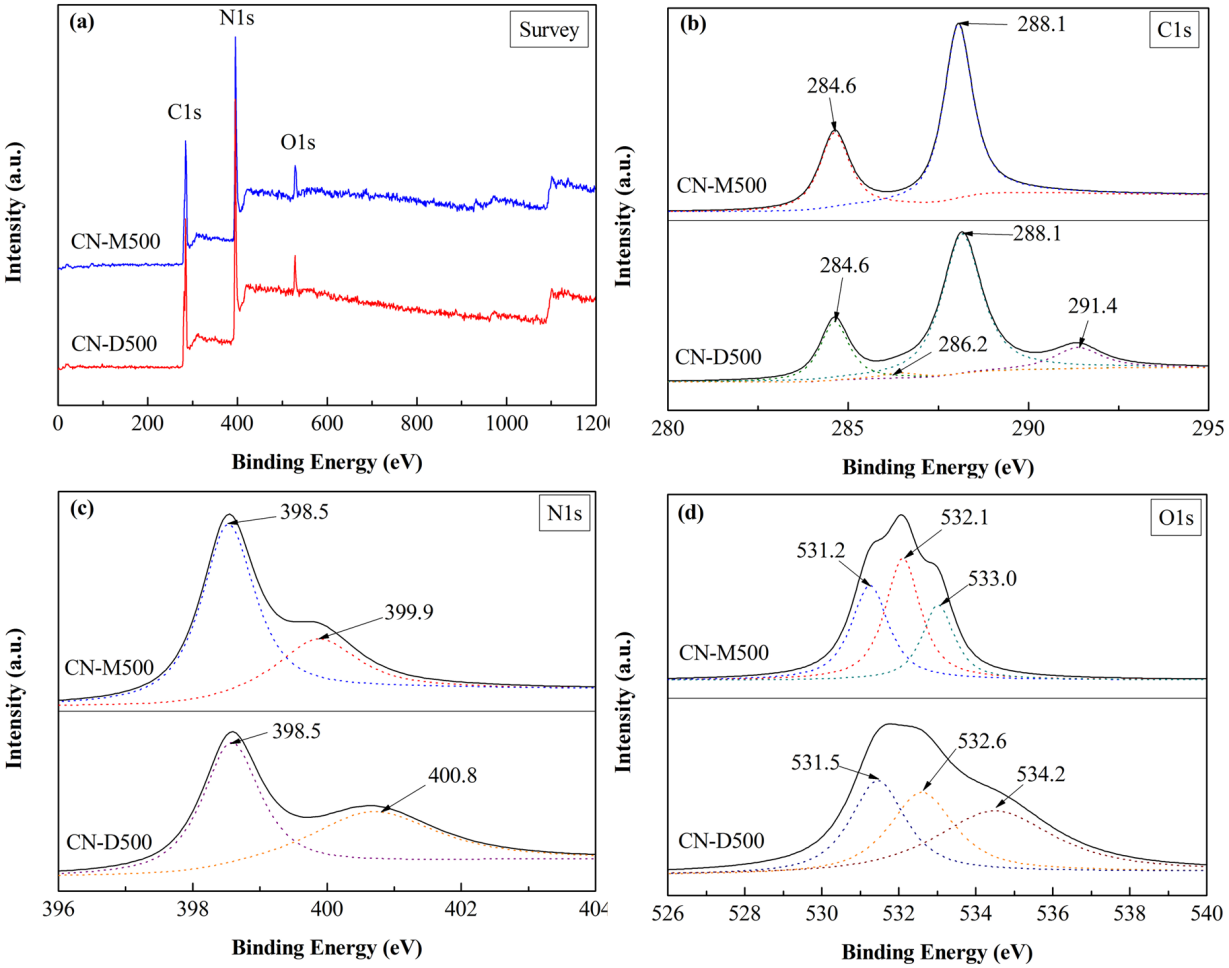
XPS spectra of CN-M500 and CN-D500. (a) survey (b) C1s (c) N1s, (d) O1s.

XPS spectra of CN-M500 and CN-D500 are shown in Fig 6. The wide-scan XPS spectra (Fig 6(a)) reveal that these two samples predominantly contain C and N elements with small amount of O element that might be attributed to the absorbed O_2_ and H_2_O or pyrolysis of the precursor in air. The atomic ratios of N/C for both samples were estimated to be 1.17 from the XPS results, a little less than the stoichiometric value. The C1s spectra in Fig 6(b) indicate that, for CN-M500, the C1s region can be fitted into two peaks, ascribed to a carbon-containing contamination (284.6 eV) and sp^2^-hybridized carbon in the aromatic ring (288.1 eV) [40]; while for CN-D500, the deconvoluted four peaks can be observed and other two peaks are assigned to C-(N)_3_ (286.2 eV) and N-C-O (291.4 eV) [33]. The formation of C-(N)_3_ and N-C-O bonds in CN-D500 might be attributed to the incomplete polycondensation of dicyandiamide precursor and redundant step for dicyandiamide to form g-C_3_N_4_. In the N1s spectra (Fig 6(c)), the main fitted peak at 398.5 eV shows the existence of sp^2^-hybridized nitrogen in C-N bonds in both samples [38]. The fitted peak at 399.9 eV in CN-M500 can be assigned to tertiary nitrogen N-(C)_3_ groups [33], and the peak at 400.8 eV in CN-D500 corresponds to -NH_2_ or = NH groups [40]. This difference also testifies the incomplete polycondensation of dicyandiamide precursor to form g-C_3_N_4_. The deconvoluted three peaks in the O1s spectra (Fig 6(d)) reveal the coexistence of chemisorbed H_2_O, O-C-N bonds and hydroxyl groups on the surface of both samples [33].

**Fig 7:**
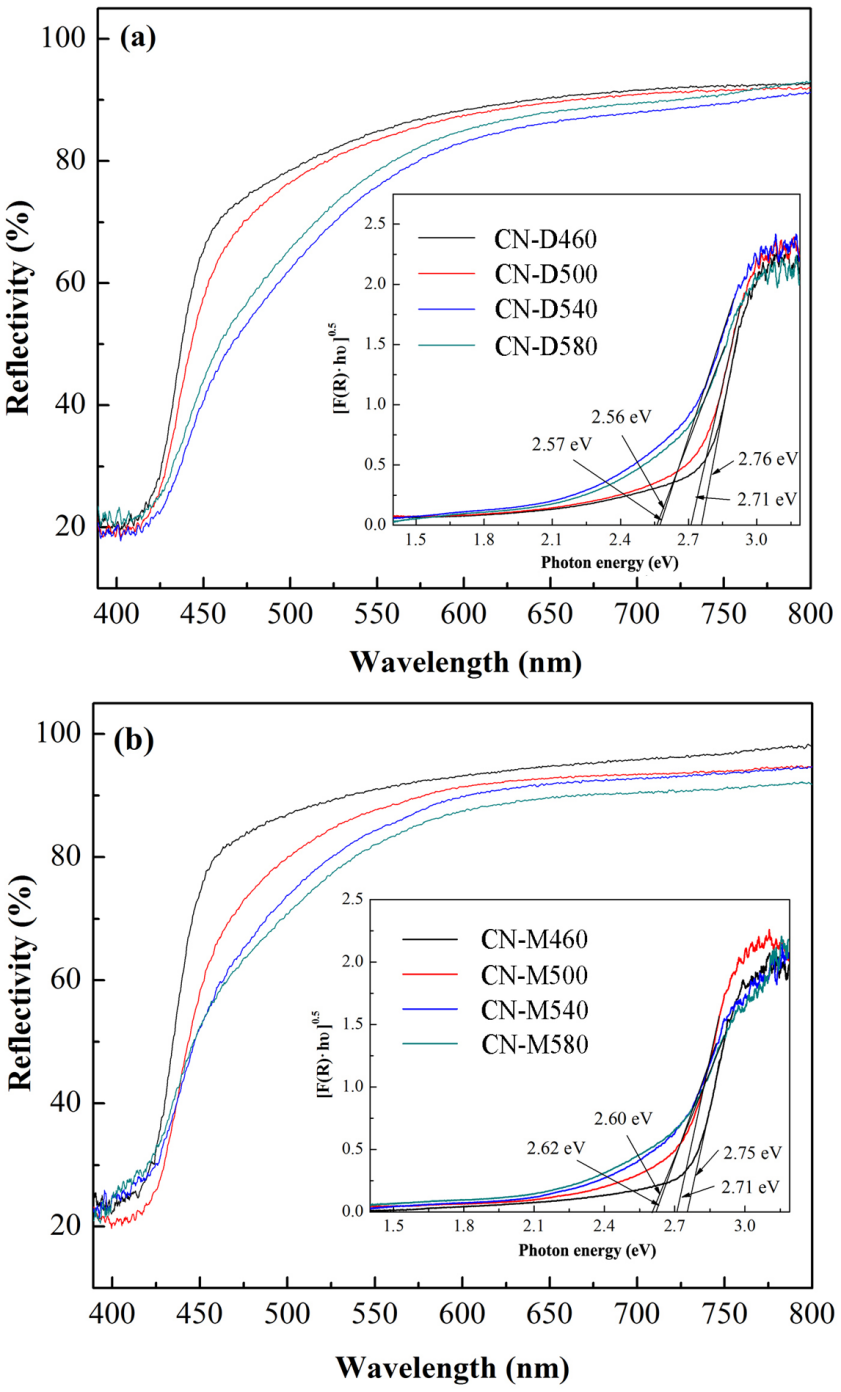
DRS spectra of g-C_3_N_4_ samples pyrolyzed from different precursors at different temperatures. The insets exhibit the relationships between [F(R)·hυ]^0.5^ and photon energy (hυ). *(a) Dicyandiamide*. *(b) Melamine*.

The DRS spectra of g-C_3_N_4_ photocatalysts derived from different precursors at different temperatures are depicted in Fig 7, where it can be found that all the samples have strong visible-light absorption edge around 450 nm, implying that all g-C_3_N_4_ photocatalysts exhibit visible-light response. Moreover, the band gap (*E*_g_) of g-C_3_N_4_ photocatalysts was determined by the method reported in previous publication [52]. As shown in the insets in Fig 7, the *E*_g_ values of g-C_3_N_4_ derived from dicyandiamide range from 2.56 eV to 2.76 eV and those from melamine range from 2.60 eV to 2.75 eV, coinciding to the literature review by Qiao’s group [53]. Moreover, it should be noted that, whether dicyandiamide or melamine was used as the precursor, the *E*_g_ value of as-obtained g-C_3_N_4_ slightly decreases with the pyrolysis temperature increasing (Table 1 and Fig 7), which is in agreement with recent report [47]. The possible explanation for this phenomenon might be that better crystallinity of g-C_3_N_4_ obtained at higher temperature make its band gap narrower.

### 3.2. Photocatalytic performance of g-C_3_N_4_ derived from different precursors

**Fig 8:**
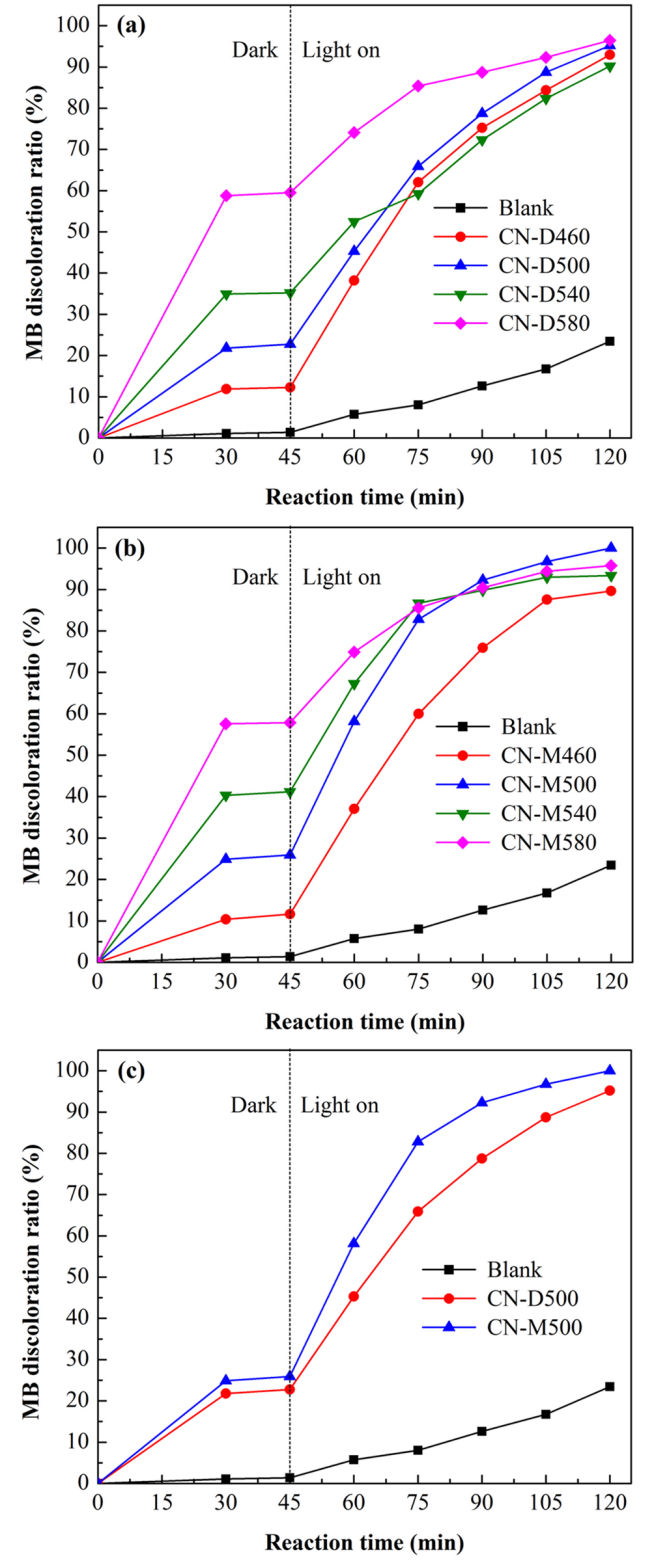
Evaluation of adsorption capacity and photocatalytic activity for g-C_3_N_4_ samples. (a) Sample derived from dicyandiamide at different temperatures (b) Sample derived from melamine at different temperatures. (c) Comparative analysis of adsorption capacity and photocatalytic activity between CN-D500 and CN-M500. All the experiments were conducted at the pristine pH value of MB solution with g-C_3_N_4_ dosage of 1.0g/L and initial MB concentration of 5mg/L.

The photocatalytic discoloration of MB over different g-C_3_N_4_ samples is illustrated in Fig 8, where it can be observed that MB can be efficiently discolored by g-C_3_N_4_ photocatalysts and the discoloration ratio of MB can reach above 90% within 120 min reaction. Blank experiments show that MB dye has a process of decomposition itself under visible light irradiation. The MB discoloration ratio by decomposition itself is near 23.5% at the irradiation time of 120 min, much lower than that by photocatalysis. Fig 8(a) clearly indicates that MB discoloration ratio is 92.9%, 95.2%, 90.2% and 96.5% for the sample CN-D460, CN-D500, CN-D540 and CN-D580, respectively, within 120 min reaction, with the highest adsorption capacity for CN-D580 and the best photocatalytic activity for CN-D500. It has been generally accepted that adsorption played an important role in the heterogeneous photocatalytic process [54]. Therefore, in this study, the specific surface area of as-obtained g-C_3_N_4_ was determined and is listed in Table 1. For the same precursor, the surface area of g-C_3_N_4_ increases when the pyrolysis temperature ascends, similar to previous report [48]. Before the photoreaction, about 12.3%, 22.8%, 35.2% and 59.5% of MB are adsorbed on the surface of CN-D460, CN-D500, CN-D540 and CN-D580, respectively, suggesting that larger sample’s surface area makes higher MB adsorption capacity. Although the adsorption capacity of CN-D580 is the highest, the reaction rate of photocatalytic discoloration driven by CN-D580 is not the fastest. The possible reason for this might be that the increase in the amount per unit area of dye molecules adsorbed onto the catalyst would reduce the number of active sites on the photocatalyst surface, which consequently hindered the generation of hydroxyl and superoxide radicals. It can be calculated that the amount per unit area of MB molecules adsorbed onto g-C_3_N_4_ sample is 3.88×10^−4^ mmol/m^2^, 3.36×10^−4^ mmol/m^2^, 5.12×10^−4^ mmol/m^2^ and 4.00×10^−4^ mmol/m^2^ for CN-D460, CN-D500, CN-D540 and CN-D580, respectively. As thus, it is not difficult to understand why the sample CN-D500 exhibits the best photocatalytic activity, instead of CN-D580. Fig 8(b) reveals a similar photocatalysis behavior of MB discoloration over g-C_3_N_4_ samples prepared from melamine at different temperatures. Likewise, the sample CN-M580 has the highest adsorption capacity and CN-M500 shows the best photocatalytic activity. Subsequently, CN-D500 and CN-M500 were selected to compare the adsorption capacity and photocatalytic activity of g-C_3_N_4_ under the identical conditions and the results are depicted in Fig 8(c). The data distinctly indicate that CN-M500 has higher adsorption capacity and better photocatalytic activity than CN-D500, attributed to its larger surface area. This result encourages us to prefer melamine to dicyandiamide as the precursor for the preparation of g-C_3_N_4_ photocatalyst. Therefore, CN-M500 was selected as the photocatalyst for the follow-up studies.

**Fig 9:**
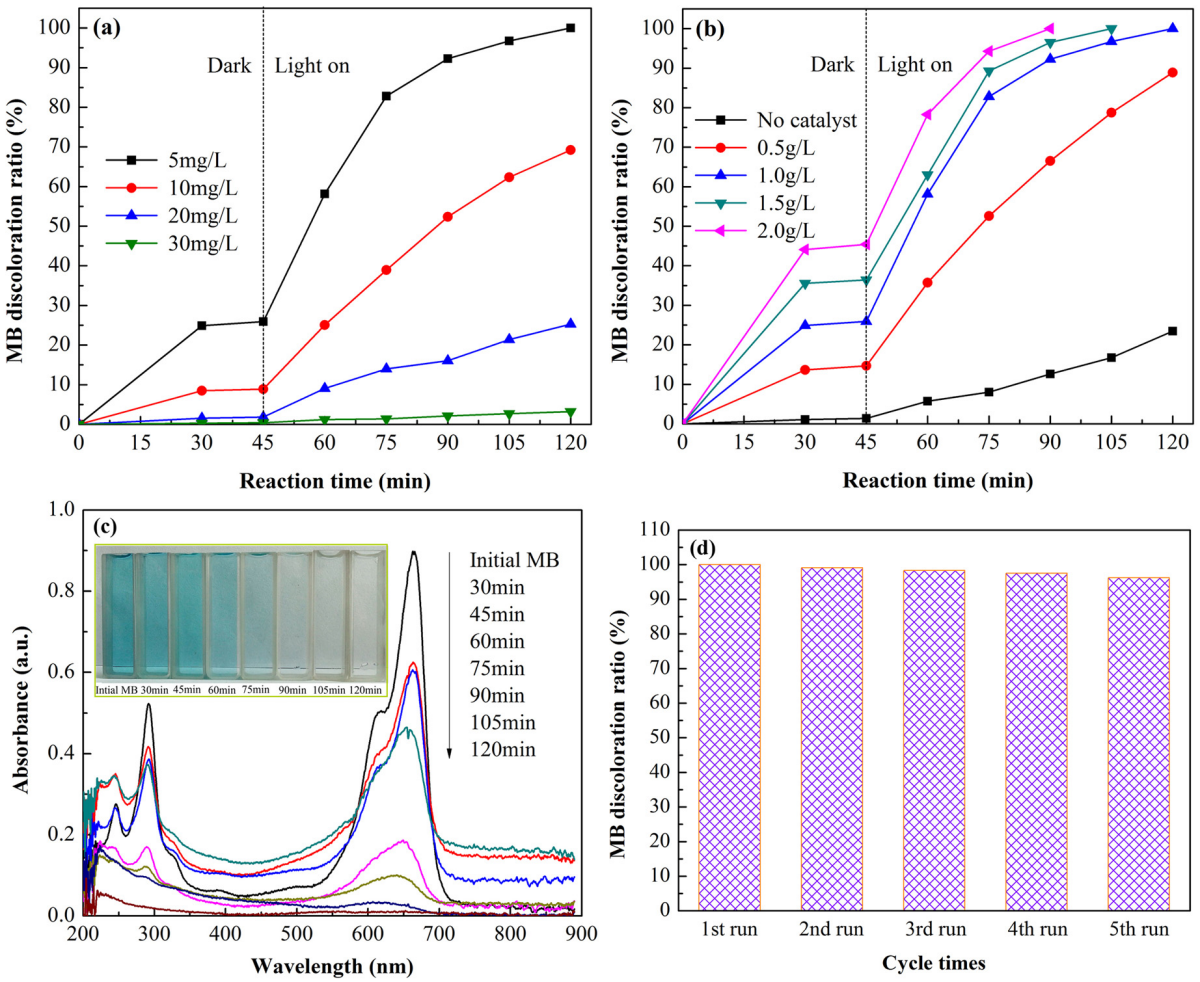
Evaluation of photocatalytic characters of CN-M500 sample. (a) Effect of initial MB concentration on the photocatalytic activity of CN-M500 at pristine pH of MB solution with g-C_3_N_4_ dosage of 1.0g/L; (b) Effect of photocatalyst dosage on the photocatalytic activity of CN-M500 at pristine pH of MB solution with initial MB concentration of 5mg/L; (c) UV-Vis absorption spectra of MB after different periods of the photocatalytic decomposition over CN-M500 at the pristine pH value of MB solution with g-C_3_N_4_ dosage of 1.0g/L and initial MB concentration of 5mg/L (inset: the color change of MB solution during reaction process); and (d) Recycle tests for CN-M500 sample under exactly identical conditions with the solid photocatalyst repeatedly washed by deionized water, centrifuged and dried after used once before.

Afterwards, the photocatalytic activity of CN-M500 was investigated under different conditions of initial MB concentration and photocatalyst dosage, as shown in Fig 9(a) and 9(b), respectively. From Fig 9(a), an obvious decrease in MB discoloration ratio from 100% to 3.2% can be observed with the increase in initial MB concentration from 5 to 30 mg/L, indicating a negative relationship between g-C_3_N_4_ photocatalytic activity and initial dye concentration. This phenomenon can be explained by the three following factors: firstly, a higher concentration of dye would reduce the active sites of g-C_3_N_4_ surface due to the adsorption of more dye molecules, hampering the formation of active oxidative species (•OH and •O_2_^-^); secondly, a higher concentration of dye would produce more intermediates with slow diffusion from catalyst surface, resulting in the deactivation of g-C_3_N_4_ photocatalyst; thirdly, a higher concentration of dye would make more light photons adsorbed by the dye itself, leading to a lower light quantum efficiency [55]. From Fig 9(b), it can be obviously found that the increase in g-C_3_N_4_ dosage can improve the adsorption capacity and photocatalytic activity for MB discoloration. When g-C_3_N_4_ dosage is 2.0g/L, MB molecules with the concentration of 5mg/L can be completely decomposed under visible-light irradiation within 90 min reaction time. This is because, with the increase in catalyst dosage, the active sites on the surface of g-C_3_N_4_ catalyst increase and generated active species increase [56]. The UV-Vis absorption spectra of MB solution after different periods of the photocatalytic decomposition are illustrated in Fig 9(c), from which it can be observed that the two main absorption peaks at 292 nm and 665 nm gradually weaken and completely disappear after 120 min reaction time, implying that MB molecules are completely decomposed into water and carbon dioxide at this moment. The inset in Fig 9(c) demonstrates that the MB solution after 120 min reaction time becomes colorless. Moreover, good stability of a photocatalyst has been regarded as another important part for the evaluation of photocatalytic activity [48]. The recyclability of CN-M500 photocatalyst was examined and the results are illustrated in Fig 9(d). After five cycles, there is no apparent decrease in MB discoloration ratio and the photocatalytic activity of g-C_3_N_4_ photocatalyst is relatively stable, suggesting that g-C_3_N_4_ photocatalyst has good stability.

### 3.3. Synergy of adsorption and photocatalysis of g-C_3_N_4_

**Fig 10:**
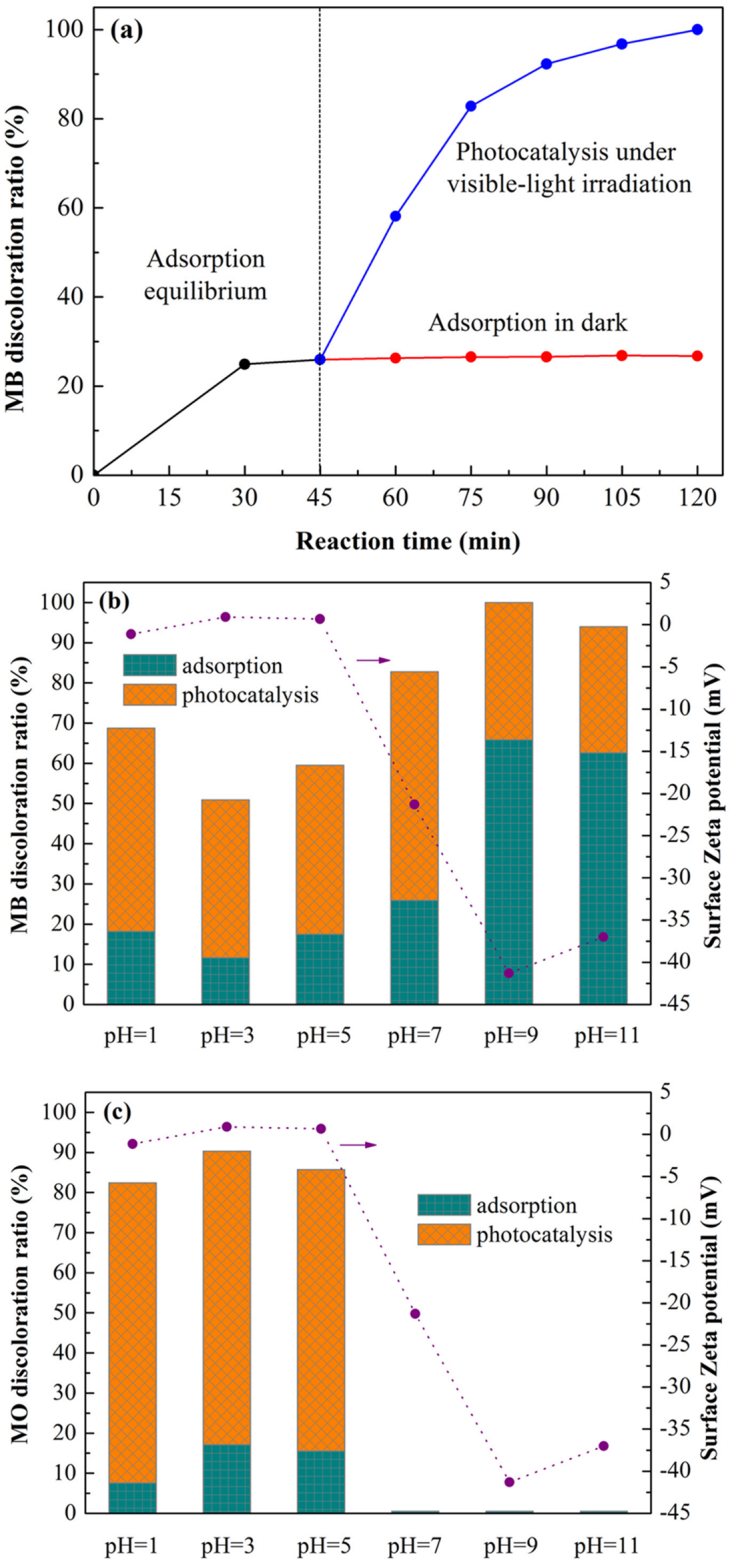
Adsorption and photocatalysis of different dyes over CN-M500. (a) Control experiments of adsorption and photocatalysis after reaching MB adsorption equilibrium with g-C_3_N_4_ dosage of 1.0 g/L and initial MB concentration of 5mg/L at pristine solution pH. Adsorption (45 min before light on) and photocatalysis (75 min after light on) of the (b) cationic dye MB and (c) anionic dye MO over CN-M500 at different medium pHs with the same g-C_3_N_4_ dosage (1.0 g/L) and initial dye concentration (5mg/L). And, the curves in (b) and (c) present the surface Zeta potentials of CN-M500 at different solution pHs.

As discussed above, adsorption and photocatalysis simultaneously occurred during MB degradation over g-C_3_N_4_. In order to further understand the relationship between adsorption and photocatalysis, several contrast tests were conducted and the surface Zeta potentials (ξ) of g-C_3_N_4_ at different pH values were measured. Firstly, the comparative experiments on MB discoloration by adsorption in dark and photocatalysis under visible-light irradiation were operated and the results are shown in Fig 10(a). For adsorption of MB onto g-C_3_N_4_ surface without visible-light irradiation, the adsorption/desorption equilibrium is established within 30 min, with nearly 25.6% MB adsorbed on g-C_3_N_4_ surface; while for photocatalysis of MB by g-C_3_N_4_ catalyst under visible-light irradiation, MB molecules in solution are almost completely decomposed within 120 min. To ensure the equilibrium of adsorption, in this study, light was turned on after 45 min adsorption in dark. Then, after a period of 75 min of photocatalysis, nearly 100% of MB was decomposed and the solution became colorless. However, the MB remained unchanged in dark within the corresponding period. Compared with adsorption only, MB discoloration ratio increased by 74.4% by photocatalysis, suggesting that it was the photocatalysis that played an important role in the complete decomposition of MB.

Naturally, a new question is proposed. What is the role of adsorption in the whole photocatalytic process? In order to answer this question, the surface Zeta potentials of CN-M500 at different pHs were measured and anionic dye MO was selected as comparison with cationic dye MB to implement another control experiment. It is well known that the surface Zeta potential of a material in solution is dependent on solution pH value. As illustrated in Fig 10(b) and 10(c), it is obvious that solution pHs have a great influence on surface Zeta potentials of CN-M500, which are -1.12 mV, 0.914 mV, 0.677 mV, -21.3 mV, -41.4 mV and -37mV at solution pH of 1, 3, 5, 7, 9 and 11, respectively. This indicates that the surface of CN-M500 possesses strong negative charges in neutral and alkaline media, weak posotive charges at pH = 3 and 5, and weak negative charges in strong acid medium, which might be attributed to the existence of multifarious functional sites on g-C_3_N_4_ surface, such as Lewis basic functions, Brönsted basic functions and H-bonding motif [27]. Moreover, it is noteworthy that the adsorption and photocatalysis of MB over CN-M500 are distinctly different from those of MO at different pHs. For MB, the amount of adsorbed dye molecules slightly decreases with the increase in pH from 1 to 3, then markedly increases with the increase in pH from 3 to 9, and finally decreases again with the increase in pH from 9 to 11. The change of MB discoloration ratio with pH is similar to that of adsorption (see Fig 10(b)). While for MO, the dye molecules can be slightly adsorbed by CN-M500 in acid media and almost no adsorption appears in neutral and alkaline media. At the same time, MO discoloration ratio is approximate to zero in neutral and alkaline media (see Fig 10(c)). On one hand, the adsorption of dye molecules is closely related to the surface Zeta potential of CN-M500, dut to the electrostatic attraction and repulsion. The surface with negative charges would enhance the adsorption of the positively charged dye molecules, such as MB; in turn, the surface with positive charges will promote the adsorption of the negatively charged dye molecules, such as MO. On the other hand, the adsorption of dye molecules facilitates their decomposition by photocatalysis. The more the adsorbed dye molecules are, the more the decomposed dye molecules are. Consequently, we have reason to believe that the adsorption is the basis and premise of photocatalysis. No adsorption, no photocatalysis. It is the synergy between adsorption and photocatalysis that is responsible for quick decomposition of dye molecules. Similar synergic effects have been proposed for other photocatalysts [57–60], while this is the first detailed study for g-C_3_N_4_ photocatalyst from the viewpoint of surface Zeta potential of photocatalyst and different types of active dye. The synergic effect can be described as follows: primarily, dye molecules are selectively adsorbed onto or near the surface of g-C_3_N_4_ photocatalyst via electrostatic attraction, leading to a higher dye concentration on or near g-C_3_N_4_ surface; subsequently, dye molecules are oxidized and decomposed on or near the surface of g-C_3_N_4_ via active species (•OH and •O_2_^-^) generated by photocatalysis; finally, the intermediate products are diffused into solution from g-C_3_N_4_ surface and dye molecules are sequentially adsorbed onto g-C_3_N_4_ surface to reach a new balance. The above three steps proceed circularly untill dye molecules are decomposed into water and carbon dioxide.

**Fig 11:**
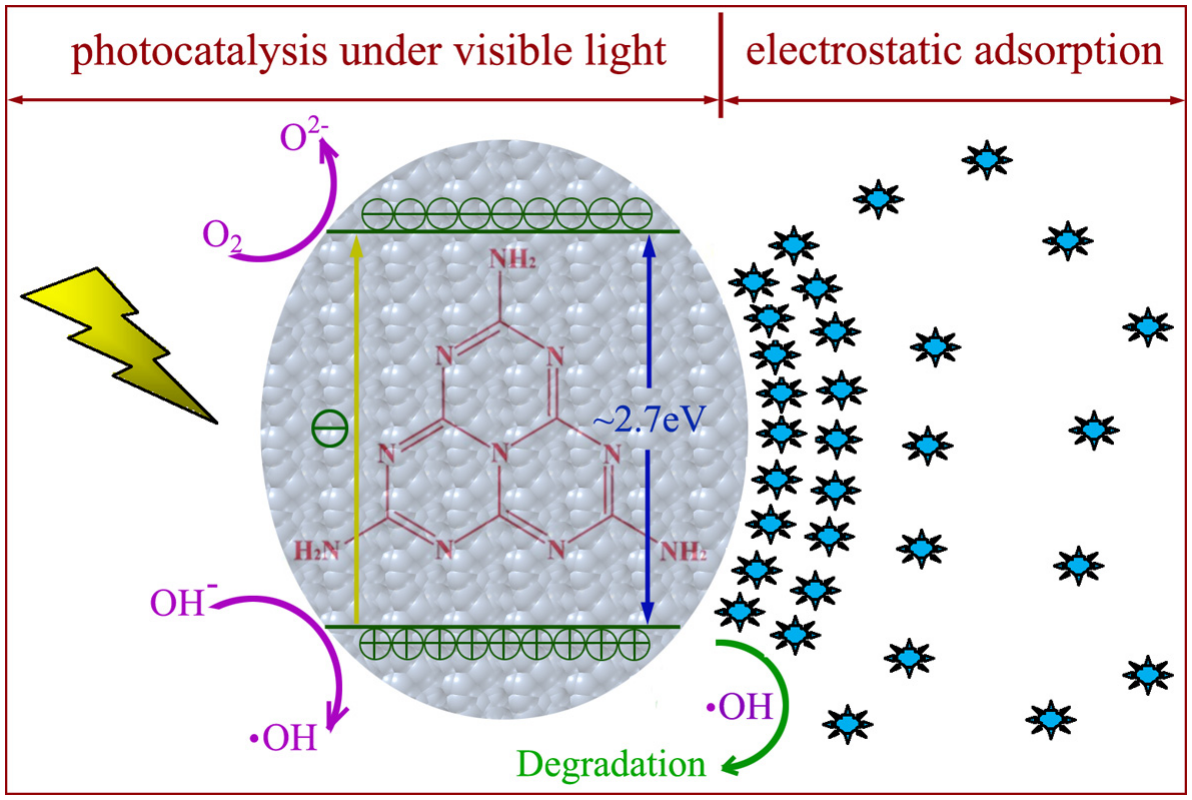
Schematic diagram for the photocatalytic degradation of dye molecules over g-C_3_N_4_ sample. The synergy between electrostatic adsorption and photocatalysis under visible light facilitates the decomposition of dye molecules.

On the whole, the possible photocatalytic process for dye discoloration over g-C_3_N_4_ is illustrated in Fig 11. At the adsorption equilibrium, large amounts of dye molecules are adsorbed on or near g-C_3_N_4_ surface due to the electrostatic attraction. The surface area and zeta potential of g-C_3_N_4_ are the two significant factors that influence its adsorption capacity. The sample with a larger surface area can be exposed to more dye molecules, and the sample with a more negative zeta potential can make more chemical interactions with dye molecules [61]. The difference in adsorption behavior of MO and MB by CN-M500 in this study might be attributed to the nature of dyes and different pHs of solution [62]. Then, under the irradiation by visible light, the electrons at valence band (+1.6 eV vs. NHE) of g-C_3_N_4_ can be migrated to conduction band (-1.1 eV vs. NHE) [63] and photogenerated electrons and holes are consequently produced. A larger surface area of g-C_3_N_4_ can provide more active sites during the photocatalytic reactions, in turn, produce more photogenerated electrons and holes [64]. Furthermore, the increased surface area can lead to less recombination of photogenerated carriers [65]. The photogenerated electrons can react with oxygen to generate superoxide radicals. The photogenerated holes can react with OH^-^ to generate hydroxyl radicals. Finally, these active radicals can make dye molecules be oxidized and decomposed. The reaction products are diffused into solution and dye molecules are sequentially adsorbed onto g-C_3_N_4_ surface to reach a new balance. The large surface area can facilitate the adsorption of reactants and diffusion of reaction products [32]. Thus, the synergy between adsorption and photocatalysis governs the quick decomposition of dye molecules. In addition to acting as an excellent photocatalyst, g-C_3_N_4_ has been developed as a potential adsorbent with superior capacity of heavy metal ions [66]. Therefore, g-C_3_N_4_ can be considered to treat the real industrial wastewater containing both organic contaminations and heavy metal ions, which would extend the practical application of g-C_3_N_4_.

## Conclusions

Pyrolytic synthesis of g-C_3_N_4_ was conducted by heating dicyandiamide and melamine, respectively, in this study. XRD results indicated that all the samples derived from different precursors at different heating temperatures possessed the same crystal structure of g-C_3_N_4_ with two diffraction peaks around 2θ of 13.10° and 27.40°. On the whole, at the same pyrolysis temperature, the crystallite size of g-C_3_N_4_ sample obtained from dicyandiamide was smaller than that from melamine. All g-C_3_N_4_ absorption bands in FT-IR spectra almost occurred at the same position for the samples obtained under different conditions. SEM observations found that all the g-C_3_N_4_ particles were in irregular shape and desultorily assembled together with the glassy morphology of typical g-C_3_N_4_. The flat layered structure could be distinctly seen in TEM images, indicating that g-C_3_N_4_ samples obtained from different precursors exhibited the same ordered structure with inter-layer stack. The lattice fringes observed in HRTEM images were assigned to (002) crystal plane and the crystal plane distances were determined as 0.323nm and 0.320nm for the g-C_3_N_4_ sample derived from dicyandiamide and melamine, respectively. The DRS spectra of g-C_3_N_4_ photocatalysts showed that all the samples had strong visible-light absorption edge around 450 nm, implying that all g-C_3_N_4_ photocatalysts exhibited visible-light response. The photocatalytic experiments indicated that MB could be efficiently discolored by g-C_3_N_4_ photocatalysts under visible-light irradiation and the discoloration ratio of MB could reach above 90% within 120 min reaction. The sample CN-M500 had higher adsorption capacity and better photocatalytic activity than CN-D500, attributed to its larger surface area. A significant decline of MB discoloration ratio happened with the increase in initial MB concentration and decrease in g-C_3_N_4_ dosage. After five cycles, there was no apparent decrease in MB discoloration ratio and the photocatalytic activity of g-C_3_N_4_ photocatalyst was relatively stable. Control experiments revealed that it was the photocatalysis that played an important role in the complete decomposition of MB. The adsorption was the basis and premise of photocatalysis and the synergic effects between adsorption and photocatalysis facilitated the decomposition of dye molecules.
